# Supplementary material for: Transmission Dynamics, Heterogeneity and Controllability of SARS-CoV-2: A Rural–Urban Comparison
Source: Int J Environ Res Public Health. 2021 May 14;18(10):5221. doi: 10.3390/ijerph18105221 (PMC8156721; doi:10.3390/ijerph18105221)
Supplement: Supplementary file 1 [file ijerph-18-05221-s001.zip › ijerph-1204942-supplementary.pdf]

Supplementary materials for "Transmission dynamics, heterogeneity, and controllability of SARS-CoV-2 in the rural area"

Yuying Li[1], Taojun Hu[1], Xin Gai[1], Yunjun Zhang[1], Xiaohua Zhou[1,2,3*]

[1] Department of Biostatistics, School of Public Health, Peking University, Beijing 100191, China

[2] Beijing International Center for Mathematical Research, Peking University, Beijing 100871, China

[3] Center for Statistical Sciences, Peking University, Beijing 100871, China

* Correspondence: azhou@math.pku.edu.cn

The plans of this supplementary material are as follows. The first section describes the three type of clusters in detail. Section 2 gives the detailed procedure of how we impute the information of transmission chains of asymptomatic cases. The following section focuses on the statistical model of constructing the likelihood. Section 4 gives two approaches to obtain the confidence interval of average reproductive number and heterogeneity parameter. Section 5 defines contact types among infections. Section 6 supplies the procedure to estimate the size of accumulated infected cases during 1 Jan 2020 and 31 March 2020. The last section provides detailed procedures in assessing the effect of vaccination for different proportions of population. Table S1 and S2 are provided at the end of the material.

1. Detailed information on three types of clusters

Simple transmission chain

A simple transmission chain has no more than two generations of cases, i.e. each chain contains only one primary case and all its secondary cases (if any). Isolated cases also belong to this type. For each simple transmission chain, we can recover the transmission history completely from the public information.

Ordinary transmission chain

An ordinary transmission chain consists of a series of transmission events in close spatio-temporal proximity. We can identify the primary case in this chain but may not recover the transmission history completely due to the low resolution of the public information. There are a lot of uncertainty of transmission relationship between inner-generations and inter-generations which hinders clarifying who infected whom. However, we may

calculate the overall size of a transmission chain, i.e. the total number of cases infected, which is easier to obtain from the public information.

Complex transmission chain

A complex transmission chain has multiple primary cases in which each of them has an equal chance to cause secondary cases. Those primary cases had similar contact history and it is hard to determine which individual is the first infected one, thus regarding them as the primary cases.

2. Imputation Mechanism

Since the individual level information of asymptomatic cases is hardly accessible, we impute this information by assuming missing at random. There are no diagnosed asymptomatic infections in urban area in our study. For the rural area, based on the constructed 655 transmission chains of confirmed infections with symptoms, the 194 asymptomatic infections are randomly allocated into those chains with a multinomial distribution for each case. The probability of each chain is proportional to the overall number of secondary cases. Without special explanation, the rest analysis is constructed based on data of imputed transmission chains, with a total of 1136 cases.

3. Constructing the likelihood

In previous studies, the average reproductive number R and the heterogeneity are mostly cared as they influence the potential size of an infectious disease collaboratively. By deploying a likelihood-based approach first proposed by,1 we can take the asymptomatic cases into consideration based on their method. Firstly, we assume the offspring of one symptomatic case follows a negative binomial distribution with size parameter equal to the heterogeneity parameter k and mean R (Table S1).[2] Without loss of generality, everyone is assumed to has the same probability of being asymptomatic, denoted by p and the asymptomatic cases have less infectiousness with a discounted average reproductive number αR, where α represents the ratio of infectiousness within symptomatic and asymptomatic cases of SARS-CoV-2, taking value of 0.26[3] in this paper. Thus, the offspring distribution of a single negative binomial distribution can be expanded to a mixture of negative binomial distribution presented by equation 1. Let S denotes the number of secondary cases infected by an arbitrary case and Y denotes the status of one individual to be asymptomatic or not taking values on $\{0,1\}$,

$$\begin{aligned} f_1(s_i;R,k) &= \sum_Y P\,(S=s_i|Y)P(Y) \\ &= p\frac{\Gamma(s_i+k)}{\Gamma(s_i+1)\Gamma(k)}(\frac{k}{\alpha R+k})^k(\frac{\alpha R}{\alpha R+k})^{s_i} \\ &+(1-p)\frac{\Gamma(s_i+k)}{\Gamma(s_i+1)\Gamma(k)}(\frac{k}{R+k})^k(\frac{R}{R+k})^{s_i} \end{aligned} \quad (1)$$

Having specifying the distribution of secondary cases, we can compute the likelihood of each type of clusters respectively based on the approach proposed by.[2] Regarding the first type of cluster, simple transmission chains, where only one primary cases transmitted to exactly one generation before the whole transmission chain die out, the likelihood can be expressed directly by a productive of offspring distribution equation 2. Suppose there are overall s_i cases in the i-th chain of simple transmission chains, $i = 1, \cdots, n_I$.

$$L_I(R,k) = \prod_{i=1}^{n_I} f_1\,(s_i - 1; R, k) \qquad (2)$$

Considering the ordinary transmission chain with one primary case and the information on the ultimate size of transmission chain, we can approximate the transmission process through a Galton-Watson branching process. Let U denotes the ultimate size of a transmission chain. According to,[1] the probability of U to be j is equivalent to $\frac{1}{j}\left(\frac{1}{(j-1)!}\frac{\partial^{(j-1)}Q(s)^j}{\partial s^{(j-1)}}\Big|_{s=0}\right)$, where $Q(s)$ is the generating function of the offspring distribution. The detailed formulation of $Q(s)$ is presented by

$$Q(s) = p\left(1 + \frac{\alpha R}{k}(1-s)\right)^{-k} + (1-p)\left(1 + \frac{R}{k}(1-s)\right)^{-k}$$

Thus, probability of a transmission chain with one primary case having an overall size of j equals,

$$\begin{aligned} f_2(j;R,k) = {} & \frac{1}{j!}\frac{\partial^{(j-1)}Q(s)^j}{\partial s^{(j-1)}}\Big|_{s=0} \\ = {} & \frac{\Gamma(kj+j-1)}{\Gamma(j+1)\Gamma(kj)}\sum_{g=0}^{j}\frac{j!}{g!\,(j-g)!}\left(p\left(1+\frac{\alpha R}{k}\right)^{-k}\right)^{g}\left((1-p)\left(1+\frac{R}{k}\right)^{-k}\right)^{j-g} \\ & \sum_{f=0}^{j-1}\frac{(j-1)!}{f!\,(j-1-f)!}\frac{B(kg+f,k(j-g)+j-1-f)}{B(kg,k(j-g))}\left(\frac{\alpha R}{\alpha R+k}\right)^{f}\left(\frac{R}{R+k}\right)^{j-1-f} \end{aligned}$$

Assume that the j-th ordinary transmission chain has an overall size of q_j, $j = 1, \cdots, n_{II}$, then the likelihood of ordinary transmission chains can be presented as,

$$L_{II}(R,k) = \prod_{j=1}^{n_{II}} f_2\,(q_j; R, k)$$

Additionally, for the complex transmission chain with more than one primary cases, we can classify these clusters into two types. As to type I complex transmission chain with known number of primary cases, denoted by m. Let T be the overall size of a complex transmission chain. According to,[4] the probability of a complex transmission chain to have an ultimate size of j equals to $\frac{m}{j}\left(\frac{1}{(j-m)!}\frac{\partial^{(j-m)}Q(s)^j}{\partial s^{(j-m)}}\Big|_{s=0}\right)$, thus the formulation of R can be presented as follows,

$$P(T=j) = f_3(j,m;R,k) = \frac{m}{j}\frac{1}{(j-m)!}\frac{\partial^{(j-m)}Q(s)^j}{\partial s^{(j-m)}}|_{s=0}$$

$$= \frac{m}{j}\frac{\Gamma(kj+j-1)}{\Gamma(kj)}\sum_{g=0}^{j}\frac{j!}{g!(j-g)!}(p(1+\frac{\alpha R}{k})^{-k})^g((1-p)(1+\frac{R}{k})^{-k})^{j-g}$$

$$\sum_{f=0}^{j-m}\frac{1}{f!(j-m-f)!}\frac{B(kg+f,k(j-g)+j-m-f)}{B(kg,k(j-g))}(\frac{\alpha R}{\alpha R+k})^f(\frac{R}{R+k})^{j-m-f}$$

As to type II complex transmission chain with unknown number of primary cases, we assume all the primary cases are transmitted by an external case. Therefore, the type II complex transmission chain with the external case comprise a complete ordinary transmission chain with only one primary case and overall size as $j+1$. Let δ denote the status of complex transmission chain to belong to type I or not, then

$$P(T=j) = \delta f_3(j,m;R,k) + (1-\delta)f_2(j+1;R,k)$$

Assume that the t-th complex transmission chain has an overall size of r_t, $t=1,\cdots,n_{III}$, then the likelihood of complex transmission chains can be presented as,

$$L_{III}(R,k) = \prod_{t=1}^{n_{III}}(\delta_t f_3(r_t,m_t;R,k) + (1-\delta_t)f_2(r_t+1;R,k))$$

The complete likelihood can be derived by a production of $L_I(R,k), L_{II}(R,k)$ and $L_{III}(R,k)$[5] and thereby the estimation of R and k can be derived by maximum the mixed likelihood function.

4. Confidence interval

Likelihood ratio test

To derive the confidence interval of R and k simultaneously, we deploy likelihood ratio test for asymptotic estimation. Traditional likelihood ratio test can be described as follows; under the null hypothesis $H_0: \theta \in \Omega_0$, the likelihood ratio asymptotically follows a χ^2 distribution,

$$\Lambda_n = \frac{\max_{\theta\in\Omega_0} L(x;\theta)}{\max_{\theta\in\Omega} L(x;\theta)}$$

$$-2\log(\Lambda_n) \sim \chi^2_{dim(\Omega)-dim(\Omega_0)}$$

Thus, let $\hat{R}$ and $\hat{k}$ denotes the maximum likelihood estimation. The corresponding confidence interval are constructed by letting $\Omega_0 = \{R \in (0,+\infty), k=\hat{k}\}$ and $\Omega_0 = \{R = \hat{R}, k \in (0,+\infty)\}$ respectively.

Biased-correlated and accelerated bootstrap

As a stochastic approach for constructing confidence interval for most complex conditions, bootstrapping plays an important role in the field of statistical inference. The basic bootstrap method, however, might perform poorly if the distribution is highly skewed. To guarantee the accuracy of confidence interval, we adopt a biased-correlated and accelerated confidence interval with R package $bootstrap$ (BC_a).[6]

5. Definition of contact type

- Primary case: The individual who was first infected in a cluster.
- Household: A household member living with a SARS-CoV-2 infected individual.
- Social: Friends, coworkers and classmates who study, work or are in close contact with the primary case.
- Community: Individual who interacts with SARS-CoV-2 infections in restaurants, entertainment venues, or other service settings.

6. Estimating the accumulative size of infected cases

Assuming the transmission follows a Galton-Watson branching process, we can reconstruct the transmission chain and predict the ongoing transmission process by simulation. The complete procedure is displayed as follows,

- According to the collected transmission chain, we define m primary cases with m transmission chains. All the subsequent infected cases belong to one of m chains.
- Set the simulation times $B = 1000$; Here we simulation 1000 times of transmission process.
- For i-th transmission chain, $i = 1, \cdots, m$, we simulate an independent branching process. The secondary cases are generated by sampling from the mixture of binomial distribution [eq1] and the infected time of secondary cases are approximated through sampling from the estimated generation time which follows a weibull distribution($\alpha = 2.015, \beta = 6.632$).
- Calculate the summation of cases in m chains who are infected before a certain date as the estimation of overall size of infection before the date.
- Repeat **Step 3-4** for B times. Assume the collected numbers are $S = q_1, q_2, \cdots, q_B$. The confidence interval is derived by taking 2.5% and 97.5% quantiles in the set S.

Thus, we can reconstruct the transmission process and predict the overall size of secondary infections by a certain date if no external infected cases are detected. To simplify the procedure of simulation, we assume all the secondary cases infected by a

single infected case have identical generation time. Nevertheless, the generation time of those infected by different cases are set to be different and sampled independently.

7. Assessment of the effect of vaccination

To evaluate the effect of vaccination, we estimate the overall size of secondary infections as of 31 Mar 2020. Assuming the efficacy of vaccination is 80%, which is equivalent to a population wide control with control effort $c = 0.8$ mentioned in.[2] The vaccination induced a population level effect identical to a reduction of average reproduction number R to $0.2R$, where the offspring distribution of one infection [eq1] is subsequently altered. The estimation of number of secondary infections is obtained by the simulation procedures illustrated in Section 6.

Additionally, we also assess the effect of vaccinating a proportion of population. Assuming the proportion of vaccination is q%, we estimate the effect of partial vaccination by randomly letting q% of transmission chains share the average average reproductive number as $0.2R$ and the average average reproductive number of rest transmission chains takes the value of R. We then estimate the overall size of secondary infection based on Section 6.

Table S1. Notations of the model

Symbol	Type	Description
i	Data	Index of simple transmission chain
s_i	Data	The number of cases in chain i
j	Data	Index of ordinary transmission chain
q_j	Data	The number of cases in chain j
t	Data	Index of complex transmission chain
r_t	Data	The number of cases in chain t
δ_t	Data	Status of complex transmission chain t to belong to type I or not
m_t	Data	The number of primary cases in Type I complex transmission chain t
f	Function	Offspring distribution
Q	Function	Generating function of the offspring distribution.
R	Parameter	Average reproductive number
k	Parameter	Dispersion parameter, $k < 1$ indicates strong heterogeneity
p	Parameter	The probability of being asymptomatic
α	Parameter	The ratio of infectiousness within symptomatic and asymptomatic cases of SARS-CoV-2

Table S2. Characteristics of the three types of transmission chains for SARS-CoV-2 outbreak in rural and urban areas

Chain type	Total number of chains (n=655)	Total number of cases (n=942)	Average chain size	Range of chain size
Rural areas				
Simple	639	751	1.2	1–5
Ordinary	15	186	12.4	3–44
Complex	1	4	4	4–4
Urban areas				
Simple	36	47	1.3	1 - 4
Ordinary	5	78	15.6	3 - 45
Complex	2	10	5	5 - 5

SARS-CoV-2, severe acute respiratory syndrome coronavirus 2

8. Reference

1. Blumberg S, Lloyd-Smith JO. Inference of r 0 and transmission heterogeneity from the size distribution of stuttering chains. *PLoS Comput Biol*. 2013;9(5):e1002993.

2. Lloyd-Smith JO, Schreiber SJ, Kopp PE, Getz WM. Superspreading and the effect of individual variation on disease emergence. *Nature*. 2005;438(7066):355-359.

3. Sayampanathan AA, Heng CS, Pin PH, Pang J, Leong TY, Lee VJ. Infectivity of asymptomatic versus symptomatic COVID-19. *The Lancet*. 2021;397(10269):93-94.

4. Blumberg S, Funk S, Pulliam JR. Detecting differential transmissibilities that affect the size of self-limited outbreaks. *PLoS Pathog*. 2014;10(10):e1004452.

5. Zhang Y, Li Y, Wang L, Li M, Zhou X. Evaluating transmission heterogeneity and super-spreading event of COVID-19 in a metropolis of china. *International journal of environmental research and public health*. 2020;17(10):3705.

6. Hall P. Theoretical comparison of bootstrap confidence intervals. *The Annals of Statistics*. Published online 1988:927-953.
